# Dual Role of LBH589 in Triple‐Negative Breast Cancer: Inhibition of Tumor Growth and Enhancement of Antitumor Immunity

**DOI:** 10.1002/cnr2.70581

**Published:** 2026-05-21

**Authors:** Liang Anjing, Yuan Rong, Xiang Su, Cheng Liang, Hou Jue, Chen Zhu

**Affiliations:** ^1^ Institute of Tissue Engineering and Stem Cells Beijing Anzhen Nanchong Hospital of Captial Medical University & Nanchong Central Hosptial, North Sichuan Medical College Nanchong Sichuan China; ^2^ Department of Surgery II Nanchong Hospital of Traditional Chinese Medicine Nanchong Sichuan China

**Keywords:** breast cancer, major histocompatibility complex I/II, panobinostat, tumor immunity

## Abstract

**Background:**

Epigenetic dysregulation, particularly aberrant histone deacetylase (HDAC) activity, plays a critical role in the progression of breast cancer. Although HDAC inhibitors (HDACi) have demonstrated antitumor potential in preclinical studies, none have been approved for breast cancer due to inconsistent efficacy across different subtypes. Panobinostat (LBH589), a pan‐HDACi with favorable pharmacological properties, has shown clinical efficacy in multiple myeloma and other malignancies, but its role in breast cancer remains insufficiently studied.

**Methods and Results:**

In this study, the effects of LBH589 on triple‐negative breast cancer (TNBC) were systematically investigated. Results revealed that LBH589 downregulated *c‐myc*, disrupted the epithelial–mesenchymal transition (EMT) program, inhibited proliferation, migration, and invasion, and induced apoptosis. Moreover, LBH589 enhanced tumor immunogenicity by upregulating MHC I/II antigen presentation pathways, thereby promoting dendritic cell maturation. In vivo, LBH589 markedly induced tumor apoptosis and inhibited growth, accompanied by increased proportions of CD8^+^ T, CD4^+^ T, and NK cells. Serum cytokine analysis showed that LBH589 significantly increased the level of IFN‐γ and decreased the levels of MCP‐1, IL‐1β, IL‐17, and IL‐10, indicating enhanced antitumor immunity and suppression of tumor‐promoting factors.

**Conclusion:**

Our study demonstrates that LBH589 not only directly induces apoptosis, inhibits proliferation, migration, and invasion, but also enhances the anti‐tumor immune response through improving tumor immunogenicity. These findings not only broaden the mechanistic understanding of HDACi in TNBC, but also provide a theoretical basis for combining epigenetic therapy with immunotherapy, supporting LBH589 as a potential immunotherapy sensitizer for triple‐negative breast cancer.

## 1 Introduction

Breast malignant tumor originates from the ductal or lobular epithelial tissue of the breast, mainly occurring in the breast glands of women. At present, breast cancer remains the leading reason of death among women aged 30–60 worldwide [1, 2]. As predicted, the proportion of breast cancer among female malignant tumors worldwide will increase to 40% until 2040 [3]. Although targeted therapies (such as HER2 targets and BRCA mutation targets) and immunotherapy have been developed, the therapeutic effect is limited due to the tumor heterogeneity of breast cancer and drug resistance [4, 5]. Because histone deacetylation (HDAC), as one of the important mechanisms of tumor progression, plays a key role in the proliferation, apoptosis, metastasis and immune escape of tumor cells, it has become a therapeutic target for many types of cancer, including breast cancer [6, 7, 8]. Currently, the histone deacetylase inhibitors (HDACi) being evaluated in clinical trials for breast cancer include Vorinostat, Belinostat, Romidepsin, Entinostat, and Panobinostat (LBH589) [9]. Regrettably, although various HDACi have exhibited anti‐tumor activity in preclinical models of breast cancer, no HDACi has been approved for the treatment of breast cancer to date [10]. It is mainly attributed to the uncertain efficacy of HDACi for breast cancer. As reported, most HDACs were found to both inhibit or promote breast cancer depending on the cell subtype. These findings indicate that the functions and targets of HDACs are not the same across all breast cancers and can depend on the subcellular location of the HDAC, breast cancer subtype, metastatic status, hormone receptor expression, and tumor grade [9]. Hence, it is of critical importance to systematically investigate the mechanism of action of HDACi in specific subtypes of breast cancer.

LBH589 is a pan‐HDACi that can inhibit HDAC I, II, and IV. It is the first HDACi approved for the treatment of relapsed or refractory multiple myeloma [11]. In addition, LBH589 is also applied in various malignant tumors such as lung cancer and lymphoma [12, 13]. Current research indicates that LBH589 exhibits superior efficacy compared to other pan‐HDAC inhibitors and is regarded as one of the most effective HDACi in current research [14]. Besides that, LBH589 demonstrates rapid oral absorption, favorable bioavailability, high patient compliance, and a lower propensity for developing drug resistance compared to other inhibitors. Compared with LBH589, Vorinostat is also orally available but has a relatively short half‐life, whereas Romidepsin and Belinostat require intravenous administration in clinic [14, 15].

**FIGURE 1:**
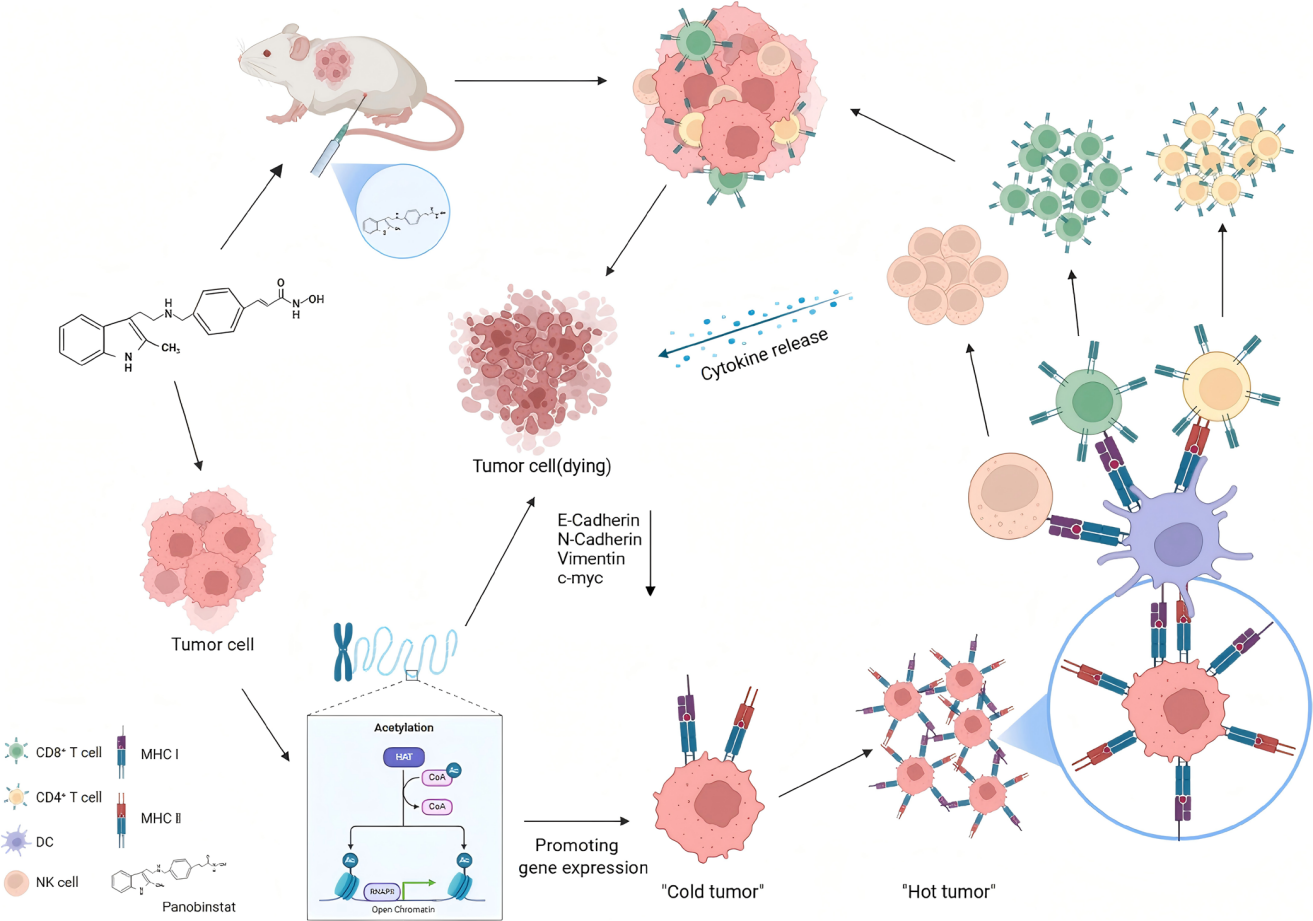
Schematic diagram illustrating the mechanism of LBH589's effect on the biological functions of triple‐negative breast cancer 4T1 cells.

In this study, we systematically evaluated the effects of LBH589 on the biological characteristics of triple‐negative breast cancer (TNBC) and the underlying mechanisms. Firstly, RNA sequencing on TNBC 4T1 cells co‐cultured with LBH589 was performed. The functionality of the differentially expressed genes was classified. These differentially expressed genes associated with cell growth, apoptosis, metastasis, and immunity of 4T1 cells were identified and validated. Our findings demonstrated that LBH589 could directly inhibit the growth of 4T1 cells by down‐regulating *c‐myc* gene expression, disputing epithelial‐mesenchymal transition (EMT), inducing apoptosis, and suppressing the migratory and invasive capabilities of 4T1 cells. Moreover, LBH589 significantly upregulates the expression of genes associated with the antigen presentation pathway, enhances the immunity of 4T1 cells, and promotes a robust anti‐tumor immune response in vivo. Overall, LBH589 effectively inhibited the growth of triple‐negative breast cancer through dual mechanisms: direct suppression of 4T1 cell proliferation and modulation of tumor‐associated immune responses (Figure 1).

## 2 Materials and Methods

### 2.1 Cell Culture

4T1 cells were purchased from the Cell Bank of Chinese Academy of Sciences (Shanghai, China). 4T1 cells were cultured in RPMI 1640 medium (Cat. no. PM150110, Pricella, Wuhan) containing 10% FBS (SFBS, Bovogen Corporation, USA), penicillin (50 U/mL) and streptomycin (50 U/mL) under conditions of 37°C and 5% CO_2_. When the cell fusion degree reaches 80%–90%, digest and passage or conduct experiments with 0.25% trypsin (Cat. no. PB180226, Pricella, Wuhan).

### 2.2 RNA Sequencing Analysis

4T1 cells (5 × 10^5^ cells) were seeded into 6‐well plates. After adherence, the medium was changed. The LBH589 group was co‐cultured with LBH589 (0.05 μM) (Catalog No. HY‐10224, MCE, USA), the control group was cultured with common medium. After co‐cultured for 24 h, cells were collected and lysed with Trizol reagent (Cat. no. 15596‐026, Invitrogen, USA). The lysed cell solution was transferred to a 1.5 mL RNase‐free Eppendorf tube for mRNA transcriptome sequencing. Total RNA from 4T1 cells was extracted using Trizol reagent. The library was sequenced through the PE150 sequencing mode (Illunima, USA) sequencing platform after passing quality inspection. Differentially expressed genes associated with tumor immunity were identified using enrichment analysis via the Gene Ontology (GO) database and Kyoto Encyclopedia of Genes and Genomes (KEGG) database.

### 2.3 RT‐qPCR

4T1 cells (5 × 10^5^ cells) were seeded into 6‐well plates. The cells were treated with the same method as described above. Then, RNA Isolation Kit (FastPure Cell/Tissue Total RNA Isolation Kit, Cat. no. RC101‐01, Vazyme, China) and a reverse transcription kit (HiScript III RT SuperMix for qPCR (+gDNA wiper), Cat. no. R323‐01, Vazyme, China) were used to extract RNA and reverse transcribe it into cDNA, respectively. The cDNA was amplified according to the instructions of SYBR qPCR Master Mix (ChamQ Universal SYBR qPCR Master Mix, Cat. no. Q711‐02, Vazyme, China). The primer sequences for RT‐qPCR are shown in Table 1. Relative expression levels of target genes were calculated by the 2^−ΔΔCt^ method, using GAPDH as an internal reference.

**TABLE 1:** RT‐qPCR primer sequences.

Genes	Forward primers	Reverse primer
*H2‐Aa*	TCCCTTCTGACGATGACATTTATGA	TTCCAGGGTGTGACTCATAAAGG
*H2‐Eb1*	GAAAGCACAATCCACATCTGCAC	TCCTTCCTTCAGCCTTGTTACTT
*H2‐D1*	TTCTGGGTGTCCTTGGAGCTATG	GAGAGACCTGAACACATCGTCTG
*CD74*	AACCATGAACAGTTGCCCATACT	GCTCACAGGTTTGGCAGATTTC
*c‐myc*	AAATCCTGTACCTCGTCCGATTC	TCTCCACAGACACCACATCAATT
*E‐cadherin*	ACTTTGGTGTGGGTCAGGAAATC	TGGCGATGATGAGAGCTACATATG
*N‐cadherin*	GGAGGAGAAGAAGACCAGGACTA	AGCAGCTTTAAGGCCCTCATTAA
*Vimentin*	CGAGAGAAATTGCAGGAGGAGAT	CTGGACATGCTGTTCCTGAATCT
*GAPDH*	CAGTGGCAAAGTGGAGATTGTTG	TCGCTCCTGGAAGATGGTGAT

### 2.4 Western Blotting (WB)

4T1 cells (5 × 10^5^) were seeded into 6‐well plates. After adherence, mediums containing different concentrations of LBH589 (0.01, 0.05, 0.10 μM) were added. In the Control group, cells were cultured with common medium. After 24 h of co‐culture, the cells were collected and centrifuged at 3000 rpm for 5 min at 4°C. The supernatant was discarded and the cells were mixed with lysate (100 mM PMSF:100 mM Protease inhibitors:RIPA lysate = 1:1:100), and the cells were lysed on ice for 30 min. The lysed solution was centrifuged at 13 000 rpm for 5 min, and the supernatant was collected to obtain the whole protein. Nuclear protein was extracted according to the instructions of nuclear protein and cytoplasmic protein extraction kit (Cat. no. P0027, Beyotime, Shanghai). The protein concentration was detected by Omni‐Easy read‐to‐use BCA Protein Quantification kit (Cat. no. ZJ102, Epizyme Biotech, Shanghai). Following SDS‐polyacrylamide gel electrophoresis, the proteins were transferred onto a nitrocellulose (NC) membrane (Catalog No. 10600002, Cytiva, Danaher Corporation). The NC membrane was blocked by immersion in PBST containing 5% skim milk powder at 4°C for 1 h. Subsequently, the blocking solution was removed, and the membrane was incubated overnight at 4°C with the following primary antibodies: anti‐H3 (1:10 000) (Catalog No. EM30605, HuaBio, China), anti‐acetyl‐histone H3 (1:500) (Catalog No. AF4365, Affinity Biosciences), anti‐α‐Tubulin (1:20 000) (Catalog No. Fnab00333, Fine Test, Wuhan), anti‐acetyl‐α‐Tubulin (1:1000) (Catalog No. AF4351, Affinity Biosciences, USA), anti‐*c‐myc* (1:500) (Catalog No. HA721182, HuaBio, China), anti‐*N‐Cadherin* (1:1000) (Catalog No. ET1607‐37, HuaBio, China), anti‐Vimentin (1:50 000) (Catalog No. ET1610‐39, HuaBio, Hangzhou), and anti‐*E‐Cadherin* (1:3000) (Catalog No. 20874‐1‐AP, Proteintech, China). The next day, after washing with PBST, horseradish peroxidase‐conjugated goat anti‐rabbit and goat anti‐mouse secondary antibodies were added and incubated for 1 h. Following another wash, the membrane was subjected to automatic imaging using a Bio‐Rad gel imager.

### 2.5 Cell Counting Kit‐8

4T1 cells (8000 cells) were inoculated into 96‐well plates. After a 24 h culture, medium contained with different concentrations of LBH589 was added. The concentrations were divided into three groups: 0.01, 0.05, and 0.10 μM. The cell survival rate was measured by CCK‐8 Kit (Cat. no. KGA317s‐500, KeyGEN Bio TECH, China) at 24, 48, and 72 h. Cell relative survival rate (%) = (OD_sample−_OD_background_)/(OD_control−_OD_background_) × 100%. Five parallel groups were used for each group.

### 2.6 Wound Healing Assay

4T1 cells (5 × 10^5^ cells) were inoculated into 6‐well plates. When the cells reached about 80% confluence, wounds were induced using a 200 μL pipette tip. Following the creation of the wounds, the wells were gently washed twice with normal saline to eliminate detached cell debris. In the control group, 2 mL of FBS‐free medium without LBH589 was added. In the LBH589 group, 2 mL of FBS‐free 1640 medium containing LBH589 was added. The cells were then incubated for 24 h. Images were captured at 0 and 24 h post‐incubation with a microscope (TS100, NIKON, Japan). Wound healing area was calculated using ImageJ with the following formula: Wound healing rate = (initial scratch width_−_final scratch width)/initial scratch width × 100%.

### 2.7 Cell Invasion Assay

Firstly, the chambers are rinsed with a medium free of FBS. Then, mixture matrix gel (Cat. no. 356234, Corning, USA) was prepared with serum‐free medium at 1:5 (V:V). Transwell chambers (Cat. no. 3422, Corning, USA) were incubated with this mixture matrix gel for 1 h. 4T1 cells suspension was washed with PBS to clear serum, and then 4T1 cells (2 × 10^4^ cells) were implanted in the upper chamber and 200 μL of medium containing FBS‐free 1640 was added. In the lower chamber, 600 μL of medium containing 20% FBS 1640 was added. In the LBH589 group, 200 μL the FBS‐free 1640 medium containing LBH589 (0.05 μM) were added into the upper chambers, and 600 μL of 20% FBS 1640 medium containing LBH589 (0.05 μM) was added into the lower chambers. After 24 h, the invasion cells were fixed with 4% paraformaldehyde for 30 min, and then stained with 0.1% crystal violet. The invasion cells were observed under Optical microscope. Under the optical microscope, three fields of view (100×) were selected for each filter membrane to count the migrating cells.

### 2.8 Cell Apoptosis

4T1 cells (5 × 10^5^ cells) were seeded into 6‐well plates. After 24 h, the medium was changed as described above. After co‐culturing with different medium for 24 h, 4T1 cells were collected, and the cells' apoptosis was measured with Annexin V/PI apoptosis kit (Cat. no. A211‐01, Vazyme, China). The cells were stained with the kit according to the instructions and analyzed by flow cytometry (FACS Calibur, BD, USA) within 1 h.

### 2.9 Tumor Immunity Assay

#### 2.9.1 The Expression of MHC I/II of Tumor Cells

4T1 cells (1 × 10^5^ cells) were seeded into 6‐well plates. The tumor cells were cultured with different medium as described above for 24, 48, and 72 h, respectively. At the predetermined time points, cells were collected and incubated with PE/Cyanine7‐mouse‐MHC I antibody (Cat. no. 116621, BioLegend, USA) and Pacific Blue‐mouse‐MHC II antibody (Cat. no. 107619, BioLegend, USA) in the dark for 30 min, centrifuged at 3000 rpm at 4°C for 10 min, and the supernatant was discarded. The cells were washed with PBS 3 times, and then analyzed by flow cytometry (NovoCyte Agilent, USA) within 1 h.

#### 2.9.2 Mature of Bone Marrow‐Derived Mesenchymal Stem Cells (BMDCs)

The leg bones of 6–8 weeks male C57BL/6 mice were harvested. The bone marrow cavity was flushed with normal saline using a syringe to collect the bone marrow suspension. The suspension was then centrifuged at 1200 rpm for 10 min, and the supernatant was carefully discarded. Subsequently, the red cells were lysed with 2–3 mL sterile ammonium chloride erythrocyte lysate (Cat. no. R1010, Solarbio, China) at room temperature for 4 min, and then the lysed reaction was neutralized with 10 mL PBS. Next, the mixture was centrifuged at 1200 rpm for 5 min, and the underlying cells were collected. The collected cells (1 × 10^6^/well) were seeded into 24‐well plate with 1640 medium containing GM‐CSF 20 ng/mL (Cat. no. 415‐ML‐010, R&D Systems, USA) and IL‐4 20 ng/mL (Cat. no. P07750, Novo Protein, China), and the medium was changed every 2 days. BMDCs were collected on 6–8 days. And 4T1 cells treated with the same method for 72 h as described above were collected. Tumor cells and BMDCs were plated (tumor cells: BMDCs = 1:5, 10^4^ tumor cells). After co‐culture for 48 h, BMDCs were collected for staining with FITC‐mouse‐CD11c (Cat. no. 117305, BioLegend, USA) and APC‐mouse‐CD80 (Cat. no. 104713, BioLegend, USA), PE‐mouse‐CD86 (Cat. no. 105008, BioLegend, USA), and analyzed by flow cytometry (NovoCyte Agilent, USA).

### 2.10 Animal Experiment

Twenty SPF grade Balb/c mice (6–8 weeks, female, 20 g) were purchased from the Animal Experimental Center of North Sichuan Medical College. All experimental protocols in this study conformed to the ethical requirements of animal experiments in North Sichuan Medical College (No. 2024073). 1 × 10^6^/100 μL 4T1 cell suspension was injected subcutaneously into the right shoulder of each mouse. When the tumor volume of mice reached 50–100 mm^3^, the mice were randomly divided into control group and LBH589 group, 5 mice in each group.

In the LBH589 group, the LBH589 were injected intraperitoneally every 2 days (15 mg/kg). The mice in the control group were injected with the equal volume of normal saline at the same time points. Tumor volume was measured every 2 days, and tumor volume was measured with the following formula, tumor volume = long diameter × short diameter × short diameter/2. The mice's weight was measured. Mice were sacrificed when the average tumor volume of the control group reached an end‐point established in our ethical procedures.

The tumor tissue were collected and the morphology and weight of tumor tissue were recorded. Some parts of the spleen and lymph nodes were prepared into single cell suspensions and stained for T cells and NK cells, including the following antibodies: Pacific Blue‐mouse‐DAPI (Cat. no. 422801, BioLegend, USA), APC‐mouse‐CD3 (Cat. no. 100236, BioLegend, USA), FITC‐mouse‐CD4 (Cat. no. 100406, BioLegend, USA), PerCp‐mouse‐CD8 (Cat. no. 100734, BioLegend, USA), and FITC‐mouse‐CD49b (Cat. no. 108905, BioLegend, USA) by flow cytometry (NovoCyte Agilent, USA) for analysis. Some tumor tissue and the main organs, including heart, liver, spleen, lung, and kidney, were removed, fixed in 4% formalin, and paraffin‐embedded sections were stained with HE.

Before sacrificed, blood was collected from the inner canthus of the mice, and the blood was collected into a 1.5 mL EP tube, centrifuged at 3000 rpm for 20 min. The supernatant was collected to obtain serum, which was tested with the LEGENDplex Mouse Inflammation Panel Kit (Cat. no. 740446, BioLegend, USA) for the detection of related inflammatory factors.

### 2.11 Statistical Analysis

A two‐tailed Student's *t*‐test was used to compare the differences between the two groups, while a two‐way multivariate analysis of variance (two‐way ANOVA) was employed to assess differences among multiple groups. A *p*‐value < 0.05 was considered statistically significant for comparisons against the control group. Each experimental group included three to five replicate samples.

## 3 Results

### 3.1 Inhibitory Effect of LBH589 on Histone Deacetylases in 4T1 Cells

**FIGURE 2:**
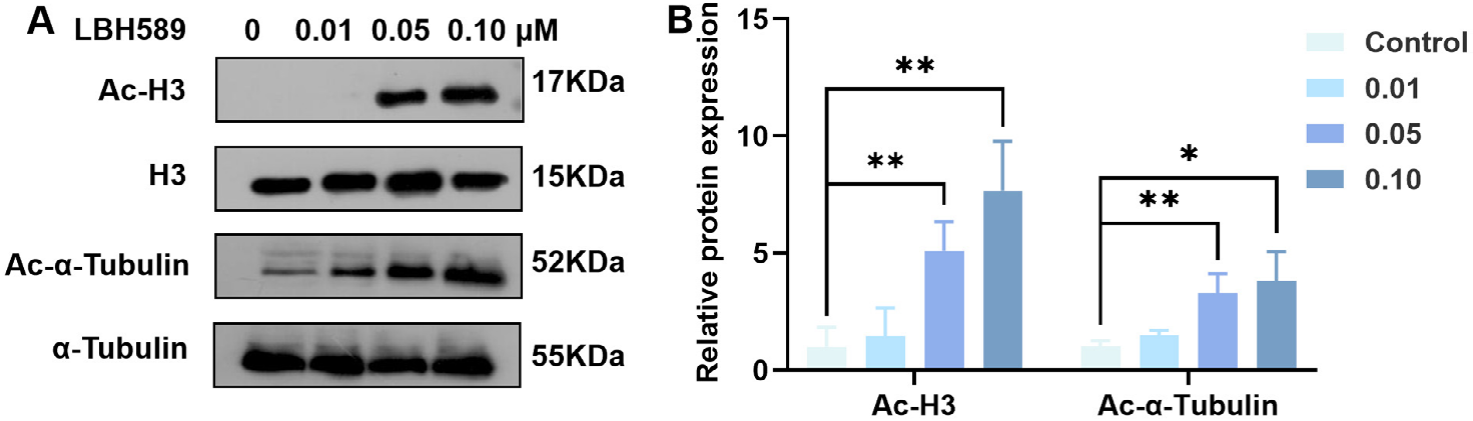
The acetylation level of nuclear histone 3 and α‐Tubulin in 4T1 cells after treatment with different concentrations LBH589. (A) WB analyses to detect acetylated; (B) statistical analysis of WB result; the raw WB images are provided in the Figure S1; *p < 0.05, **p < 0.01, *n* = 3.

To determine the effective concentration of LBH589 in breast tumor cells, 4T1 cells were co‐cultured with different concentrations of LBH589 for 24 h. As shown in Figure 2A,B, S1, LBH589 significantly induced the acetylation of histone (H3) and α‐Tubulin on 4T1 cells (*p* < 0.05). The acetylation of significantly increased indicated that 4T1 cells were sensitive to LBH589.

### 3.2 mRNA Sequencing Results

**FIGURE 3:**
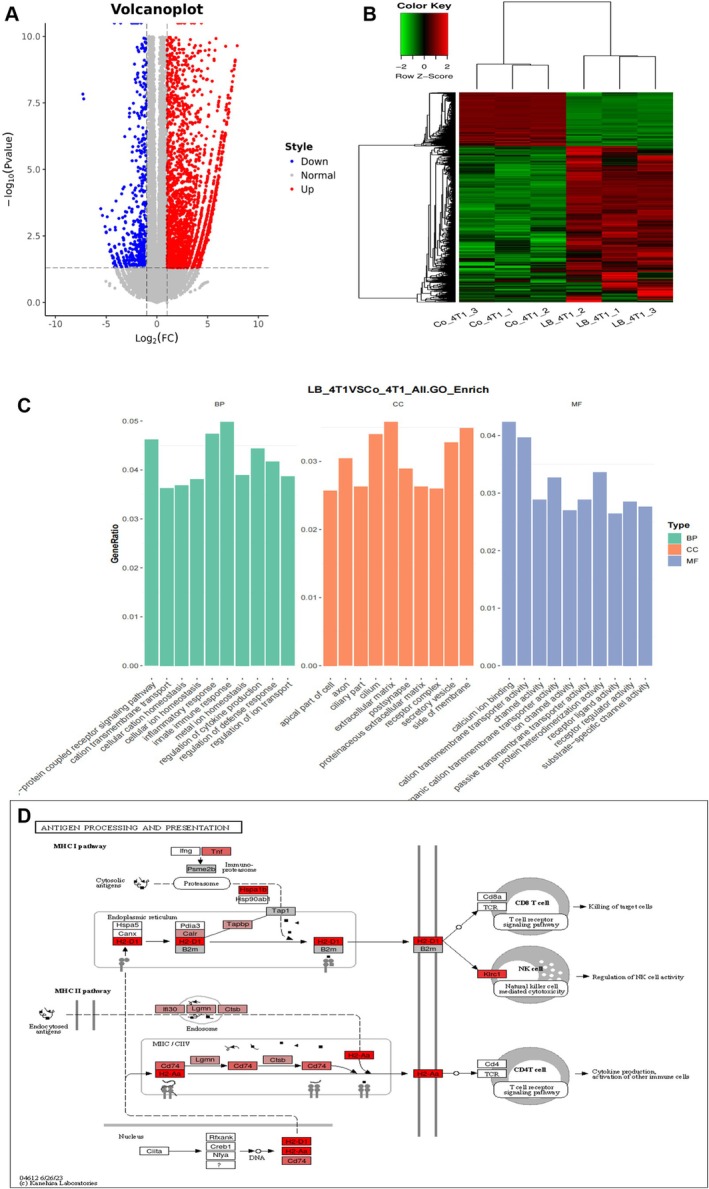
mRNA sequencing results of 4T1 cells treated with LBH589. (A) Volcano plot of differential genes; (B) cluster heatmap of differentially expressed genes; (C) functional analysis of differential genes in GO database; (D) analysis of the KEGG database indicated that a substantial number of differentially expressed genes were significantly enriched in signaling pathways associated with the antigen presentation pathway.

To explore the mechanism of LBH589, this study evaluated its impact on the gene expression of 4T1 cells using mRNA sequencing analysis. The mRNA sequencing results showed that there were 5319 upregulated genes and 1210 downregulated genes following treatment with LBH589 (Figure 3A). Functional analysis of these differentially expressed genes indicated that LBH589 displayed a significant influence on the immune response of 4T1 cells. GO database analysis and cluster analysis revealed that these genes were predominantly involved in innate immune processes (Figure 3B,C). Furthermore, KEGG pathway enrichment analysis identified significant alterations in multiple genes associated with antigen presentation, including *H2‐Aa*, *H2‐Eb1*, *H2‐D1*, and *CD74* (Figure 3D). Notably, several genes closely linked to tumor phenotypes also exhibited differential expression, such as *c‐myc*, *E‐cadherin*, *N‐cadherin*, and *Vimentin*.

### 3.3 LBH589 Affects the Cell Phenotype of Breast Tumor Cells

#### 3.3.1 Effect of LBH589 on the Proliferation of Breast Cancer

**FIGURE 4:**
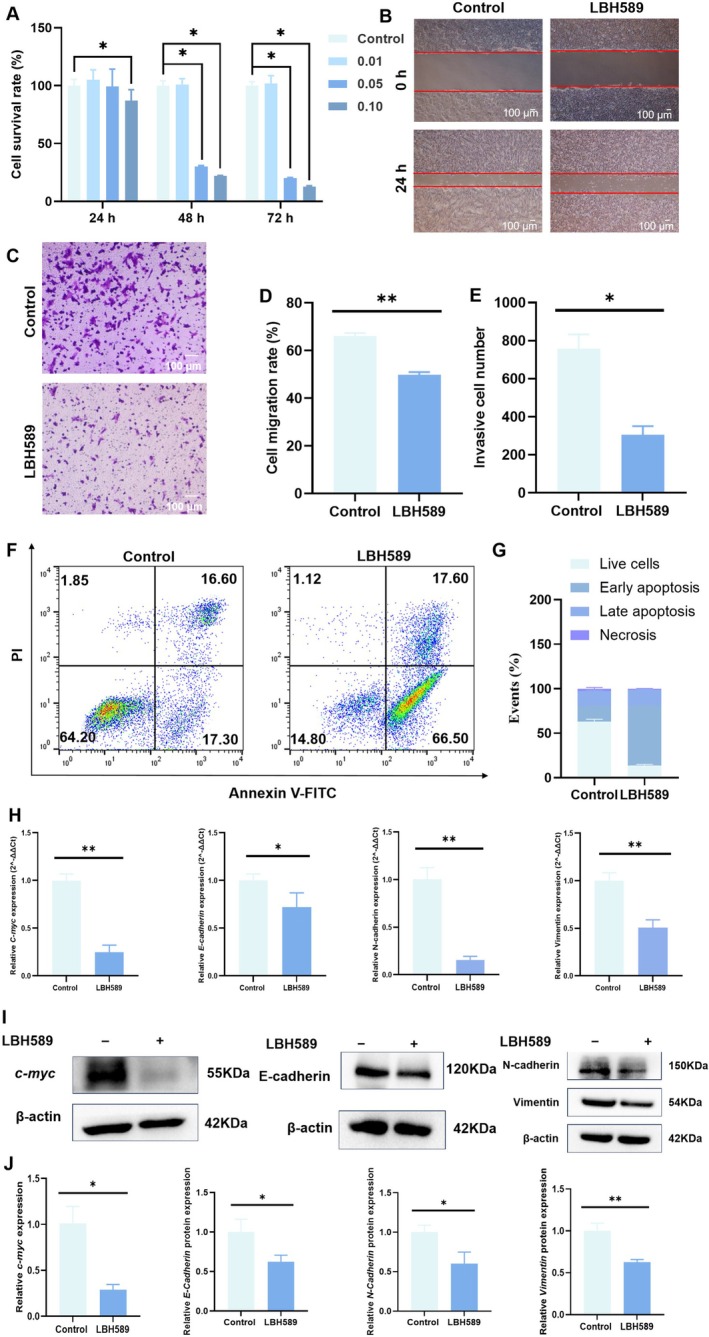
Effects on the phenotype of 4T1 cells after treatment with LBH589. (A) The proliferation of 4T1 cells after treatment with different concentrations of LBH589, *n* = 5; (B, C) representative images of influence on migration (B) and invasion (C) ability of 4T1 cells after treatment with different concentrations of LBH589, scale bar = 100 μm; (D, E) statistical results of influence on migration (D) and invasion (E) ability of 4T1 cells after treatment with different concentrations of LBH589; (F, G) representative flow cytometry images (F) and statistical results (G) of apoptosis of 4T1 cells after treatment with different concentrations of LBH589; (H) RT‐qPCR analysis of changes in the expression of related genes; (I, J) representative WB results (I) and statistical results (J) of relative different genes of 4T1 cells after treatment with different concentrations of LBH589, the raw WB images are provided in the Figures S2 and S3; *p < 0.05, **p < 0.01, *n* = 3.

The effect of LBH589 on the proliferative activity of 4T1 cells was evaluated by CCK‐8. The results showed that LBH589 significantly inhibited the growth of 4T1 cells in a dose‐dependent manner (Figure 4A). Only when the concentration of LBH589 was up to 0.05 μM, the growth of tumor cells was significantly inhibited. At 48 h, the survival rates were 30.29% ± 0.78% and 22.01% ± 0.38% in the 0.05 μM group and 0.10 μM group, respectively. At 72 h, the survival rates were 20.25% ± 0.46% and 12.83% ± 0.79% in the 0.05 μM group and 0.10 0.10 μM group, respectively.

#### 3.3.2 Effect of LBH589 on Migration and Invasion of Breast Tumor Cells

As shown in Figure 4D, the migration of 4T1 cells was obviously affected by LBH589. The wound healing rate of 4T1 cells in the control group and LBH589 group was 66.11% ± 1.29% and 49.76% ± 1.18%, respectively. The effect of LBH589 on breast cancer invasion was also observed by analyzing the number of 4T1 cells that passed through the Transwell chamber after LBH589 treatment. As shown in Figure 4E, the invasive cells number decreased from 778 ± 28 to 302 ± 9 after the treatment of LBH589 (*p* < 0.05). These data suggested that LBH589 could significantly inhibit the migration and invasion ability of breast tumor cells. Then, the related EMT gene and protein were investigated through RT‐qPCR and WB (Figure 4H–J, S2). These data showed that the genes and protein expression levels of *N‐Cadherin*, *E‐Cadherin*, and *Vimentin* markers associated with EMT were significantly lower in the LBH589 group compared to those in the control group (*p* < 0.05).

#### 3.3.3 Effect of LBH589 on Apoptosis on Breast Cancer

Furtherly, the effect on the tumor cell apoptosis of LBH589 was measured. As shown in Figure 4F,G, the early apoptosis rate and late apoptosis rate of 4T1 cells in the control group were 17.17% ± 0.32% and 16.83% ± 0.25%, respectively. Whereas the early and late apoptosis rates of 4T1 cells in LBH589 group were 66.97% ± 0.42% and 18.33% ± 0.15%, respectively, which were significantly higher than those in the Control group (*p* < 0.05). The results indicated that LBH589 can promote the apoptosis of breast tumor cells. RT‐qPCR showed that the relative gene expression of *c‐myc*, which is related to cell proliferation and apoptosis, was significantly decreased in 4T1 cells, and WB showed that the expression of *c‐myc* protein was also significantly decreased (*p* < 0.05) (Figure 4H–J, S3).

#### 3.3.4 Effect of LBH589 on the Breast Tumor Immunity

**FIGURE 5:**
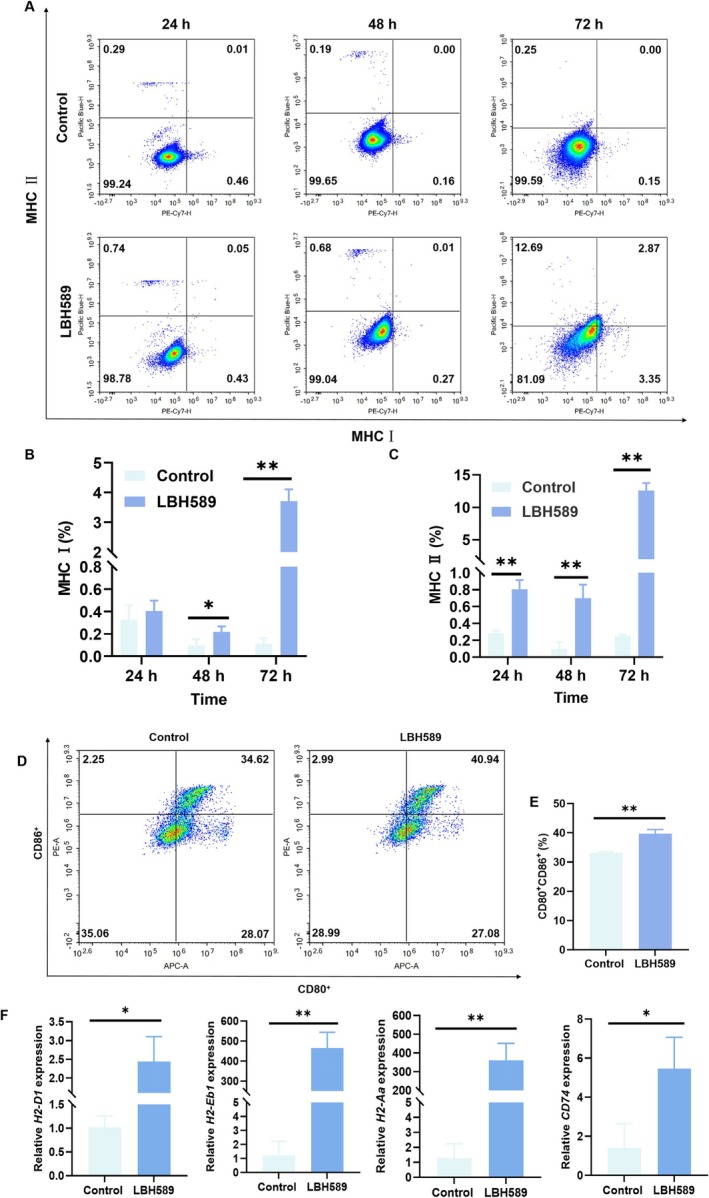
Change in the immunogenicity of 4T1 cells after treated with LBH589. (A–C) Representative flow cytometry images (A) and statistical results (B, C) of the express of MHC I/II expression on 4T1 cells; (D, E) Representative flow cytometry images (D) and statistical results (E) of mature BMDCs after co‐cultured with 4T1 cells; (F) RT‐PCR analysis of changes in the expression of antigen presentation pathway‐related genes. *p < 0.05, **p < 0.01, *n* = 3.

Order to explore the effect of breast tumor immunity, the expression of MHC I/II was measured after treated with LBH589. As shown in Figure 5A–C, MHC I/II on 4T1 cells was also significantly increased after co‐culture with LBH589. The expression of MHC I and MHC II in LBH589 group increased from 0.10% ± 0.06% to 0.22% ± 0.05% and 0.10% ± 0.08% to 0.70% ± 0.16% at 48 h, respectively. The expression of MHC I increased from 0.11% ± 0.05% to 3.71% ± 0.40% and MHC II increased from 0.25% ± 0.02% to 12.59% ± 1.15% at 72 h (*p < 0.05*). Furtherly, the tumor cells treated with LBH589 were co‐cultured with BMDCs to explore whether it could promote the maturation of BMDCs. The results showed that the ratio of CD80^+^/CD86^+^ cells increased from 33.11% ± 0.41% to 39.71% ± 1.45% when 4T1 cells were co‐cultured with BMDCs (1:5) (*p* < 0.05) (Figure 5D,E). These data shows that LBH589 can increase the expression of MHC I/II molecules on the surface of 4T1 cells, promote the maturation of BMDCs, facilitate the presentation of antigen‐specific signals to T cells, and activate T cells to kill tumor cells. LBH589 can significantly improve the immunity of 4T1 cells.

To investigate the mechanism by which LBH589 influences the immune response of breast cancer cells, we examined the expression levels of relevant genes. As shown in Figure 5F, RT‐qPCR results showed that the expression of some antigen presenting related genes (*H2‐Aa, H2‐Eb1, H2‐D1, CD74*) was significantly up‐regulated in the LBH589 group (*p* < 0.05). The upregulation of these genes may be the main reason contributing to the increased expression of MHC on the surface of breast tumor cells.

### 3.4 Animal Study

#### 3.4.1 Tumor Inhibition Assay of LBH589 in Tumor‐Bearing Mice

**FIGURE 6:**
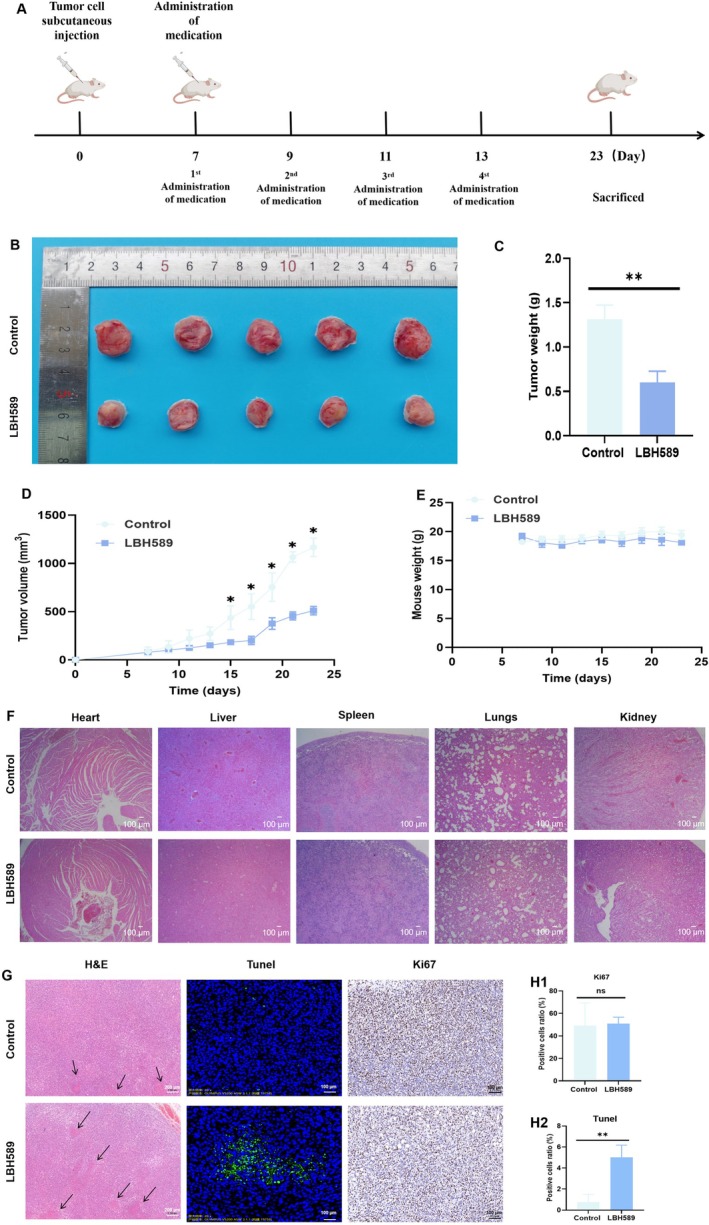
In vivo anti‐tumor efficiency analyses of LBH589. (A) The schematic of experiment design; (B) the mass observation of tumor tissue after treated with LBH589; (C) the tumor tissue weight at 23th day; (D, E) the tumor growth curve (D) and mice weight (E), *n* = 5; (F) the results of H&E staining results of the main organ (scale bar = 100 μm); (G) the H&E staining(scale bar =200 μm), tunel staing and Ki67 staining of tumor tissue (scale bar = 100 μm), *n* = 3, black arrows shows the necrotic tissue; (H) the quantitative calculation of the positive cells ratio of Ki67 (H1) and Tunel (H2) staining of tumor tissue; **p < 0.01, *n* = 5.

As shown in Figure 6, LBH589 can significantly inhibit tumor growth. On the 23rd day, the average tumor volume in the control group reached 1167.11 ± 95.82 mm^3^, whereas in the LBH589 group, it was significantly reduced to 511.12 ± 43.74 mm^3^. The tumor volume is approximately half of that in the control group. The tumor weight exhibited a similar trend to the tumor volume, with the control group showing an average weight of 1.23 ± 0.21 g and the LBH589 group demonstrating a markedly lower weight of 0.60 ± 0.13 g (*p* < 0.05) (Figure 6A–E). The H&E staining results of the main organs showed that there were no significant structural changes and inflammatory responses in the main organs of the mice in the LBH589 group(Figure 6F). These data indicated that the dosage of LBH589 employed in this study is in the safe range, without inflicting any substantial adverse effects on the major organs. The results of H&E staining and TUNEL staining indicated that the tumor tissue exists with more necrotic tissue and apoptotic cells in the LBH589 group (Figure 6G). As shown in Figure 6, the proportion of apoptotic cells in the tumor tissues of the LBH589 group was 5.02% ± 1.16%, whereas that in the control group was only 0.77% ± 0.72% (*p* < 0.05). The Ki67 staining results showed that the treatment with LBH589 had no significant effect on the Ki67 index. The in vivo antitumor effect of LBH589 was consistent with the results obtained from the in vitro cell experiment.

#### 3.4.2 Regulation of Anti‐Tumor Immunity by LBH589 in Tumor‐Bearing Mice

**FIGURE 7:**
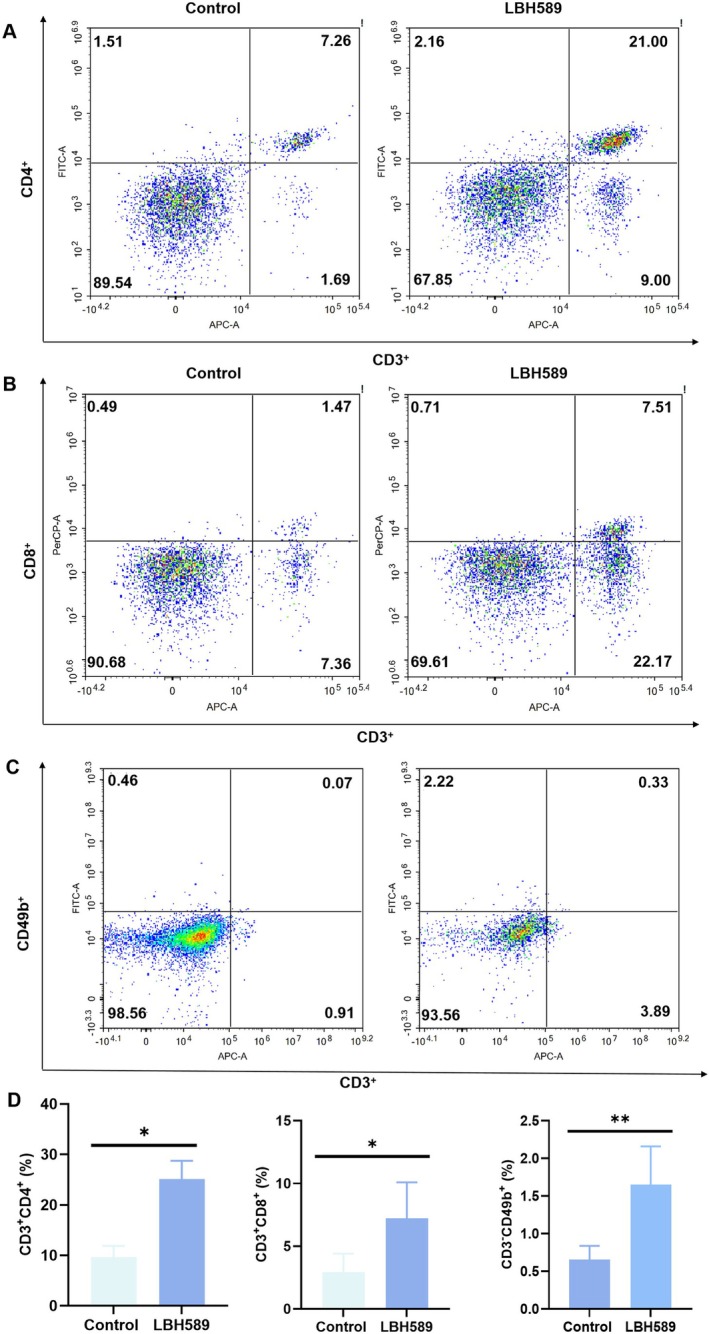
The status of immune cells in tumor‐bearing mice following treatment with LBH589. (A, B) Representative flow cytometry images of CD3^+^CD4^+^ T cells and CD3^+^CD8^+^ T cells in lymph node; (C) representative flow cytometry images of NK cells in spleen; (D) statistical results of flow cytometry results in vivo. *n* = 3, *p < 0.05.

To further investigate whether LBH589 could activate anti‐tumor immune responses in vivo, single cell suspensions of lymph nodes and spleens were obtained and stained for T cells and NK cells. As shown in Figure 7, LBH589 significantly increased the proportion of CD8^+^ T cells and NK cells with anti‐tumor effect, which indicates LBH589 effectively activated the body's anti‐tumor immune response. In Balb/c mice, the percentage of CD4^+^ T cells in lymph nodes increased from 9.63% ± 2.26% to 25.14% ± 3.60% and the percentage of CD8^+^ T cells increased from 2.94% ± 1.47% to 7.22% ± 2.88%. The percentage of NK cells in spleen increased from 0.66% ± 0.18% to 1.65% ± 0.51% (*p* < 0.05) (Figure 7).

**FIGURE 8:**
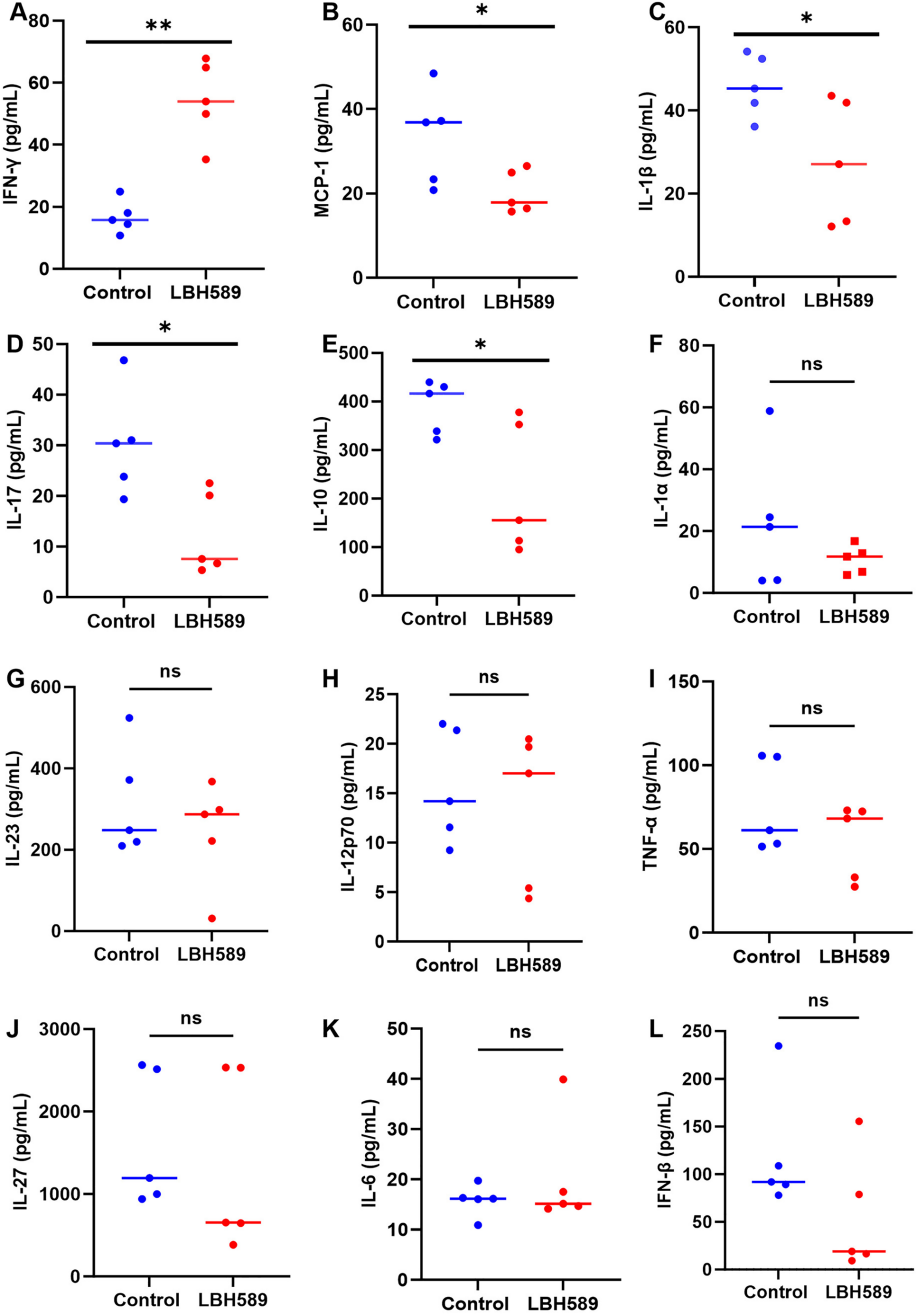
Change of inflammatory cytokines in serum in each groups. *p < 0.05, **p < 0.01 *n* = 3.

Furtherly, the inflammatory in serum was tested by CBA Kit. As shown in Figure 8, the concentration of interferon‐γ (IFN‐γ), a classic cytokine with anti‐tumor function, was significantly higher in the LBH589 group at 54.41 ± 13.00 pg/mL compared to the control group, in which the concentration was 16.78 ± 5.24 pg/mL (*p* < 0.05). Except that, the concentration of monocyte chemoattractant protein‐1 (MCP‐1), interleukin‐1β (IL‐1β), interleukin‐17 (IL‐17), and interleukin‐10 (IL‐10) in the LBH589 group was all decreased (*p* < 0.05) (Figure 8).

## 4 Discussion

The progression of breast tumor is closely linked to epigenetic alterations, including histone acetylation and DNA methylation, which are recognized as key regulatory mechanisms in carcinogenesis [16, 17]. As reported, decreased expression of tumor‐suppressor genes is significantly associated with the upregulation of HDACs in patients diagnosed with breast cancer [18]. According to references, HDAC2, HDAC3, HDAC6, HDAC8, and HDAC9 are all markedly overexpressed in breast cancer tissues [19]. Consistent with its role as a pan‐HDAC inhibitor, LBH589 treatment led to a marked increase in histone acetylation of H3, indicating effective inhibition of class‐I HDAC activity(e.g., HDAC1, HDAC2, HDAC3). At the same time, a significant increase acetylation of α‐tubulin is also observed (Figure 2). α‐tubulin is a well‐established substrate of HDAC6, demonstrating concomitant inhibition of class‐II HDAC activity.

HDACs induce chromatin condensation by eliminating acetyl groups from histones. This biochemical process directly influences the transcriptional activities of tumor suppressor genes, apoptosis regulators, and factors involved in tumor metastasis. In this study, it was found that LBH589 could significantly induce 4T1 cells apoptosis and inhibit the proliferation of 4T1 cells, suggesting its potential value in the treatment of breast cancer (Figure 4A,F,G). The results of RT‐PCR and WB demonstrated that after treatment of 4T1 cells with LBH589, both the gene expression and protein expression of *c‐myc* were significantly downregulated (*p* < 0.05, Figure 4H–J). It may be the key pathway by which it induces apoptosis and suppresses tumor growth. *c‐myc* is a recognized oncogene that is highly expressed in multiple tumor types and is involved in regulating numerous biological processes, including the cell cycle, metabolism, transcription, and apoptosis. Its abnormal activation is often closely associated with the proliferation, metabolic reprogramming, and anti‐apoptotic properties of tumor cells [20, 21]. As reported, HDACi may inhibit the expression of *c‐myc* by modulating the epigenetic state of the chromatin regions associated with *c‐myc*. It means HDACi primarily downregulates *c‐myc* expression through inhibition of class‐I HDAC activity [22]. As shown in Figure 2, a significant increase in Ac‐H3 was observed, indicating effective inhibition of class‐I HDAC activity by LBH589. Therefore, the downregulation of *c‐myc* expression in 4T1 cells may be attributed to the inhibition of class‐I HDAC activity.

Metastasis is a critical hallmark of malignant tumor progression, hence, the effects and the potential molecular mechanisms of LBH589 on the migration and invasion capacities of the highly metastatic murine breast cancer cell line 4T1 were evaluated. As reported, HDAC6 can affect cell adhesion and migration by modulating cytoskeletal dynamics, thereby fostering tumor metastasis and invasion. In breast cancer cells exposed to low shear stress conditions, HDAC6 facilitates directional cell migration through the deacetylation of microtubules [23, 24]. Hence, the significant increase of Ac‐α‐Tubulin may be an important reason for the significant inhibition of the migration and invasion abilities of 4T1 cells (Figure 2). On the other side, 4T1 cells inherently exhibits a robust EMT phenotype, which confers significant migratory and invasive abilities. Key molecular features of this EMT phenotype include the high expression of *N‐Cadherin*, *Vimentin* and the low expression of *E‐Cadherin*. Our RT‐PCR and WB results revealed that the expression levels of *N‐Cadherin* and *Vimentin* in 4T1 cells were significantly reduced following treatment, whereas *E‐Cadherin* expression did not show a notable recovery and even exhibited a further decline(Figure 4H–J). Recent studies, especially those based on single‐cell sequencing, have revealed that EMT is not a binary process, but a heterogeneous and dynamic disposition with intermediary or partial EMT states [25, 26, 27, 28]. Tumor cells can exist in intermediate epithelial/mesenchymal (E/M) states, commonly referred to as partial EMT or hybrid E/M phenotypes. Hence, the downregulation of mesenchymal markers may reflect that the EMT program has been disrupted by LBH589. The decrease expression of *E‐Cadherin* may be attributed to the regulation of *E‐cadherin* involves multiple epigenetic layers, including DNA methylation and transcriptional repression, which may not be reversed by HDAC inhibition alone. Further investigation into the expression of key EMT/MET transcription factors (e.g., *Snail*, *Zeb1*, *Ovol2*) and the re‐establishment of cell–cell junctions will be necessary to confirm whether this represents a true MET transition.

In recent years, immunotherapy, which regulates the immune function to exert the immune system's surveillance role on tumor cells, has brought a promising hope for the treatment of patients with advanced tumors. At present, immunotherapies, such as immune checkpoint inhibitors, tumor vaccines, and adoptive T cells have been applied in the treatment of advanced or metastatic breast tumor [29]. However, due to the fact that breast cancer is characterized by a low mutational burden, low immunogenicity, and an immunosuppressive tumor microenvironment, it is generally classified as a “cold” tumor with a limited immune response rate [30]. Although some immunotherapies have been explored for the treatment of breast cancer, clinical data indicate that a substantial proportion of patients remain nonresponsive. Keynote trial series have demonstrated promising efficacy in TNBC. But approximately 40%–50% of patients still fail to respond to these immunotherapies [31, 32]. In addition, patients with heavily pretreated or advanced TNBC often exhibit a more immunosuppressive tumor microenvironment and reduced responsiveness to immune checkpoint blockade, highlighting an urgent need for strategies that enhance tumor immunogenicity [33]. Therefore, immunotherapy in breast tumor is limited in clinic. Thus, improving the immunogenicity of breast cancer and enhancing the response rate of immunotherapy will help more patients benefit from immunotherapy. In this study, mRNA sequencing indicated that 4T1 cells treated with LBH589 could activate the MHC I/II signaling pathway. MHC I/II are two important antigen presentation pathways, mediating the activation of CD8^+^ T and CD4^+^ T cells respectively [34, 35]. It is widely accepted that MHC I are expressed on the surface of most nucleated cells and are recognized by CD8^+^ T cells, whereas MHC II are predominantly expressed on antigen‐presenting cells and play a role in helper T cell recognition. In recent years, increasing evidence has demonstrated that tumor cells can acquire antigen‐presenting capabilities through the aberrant activation of the MHC‐II expression pathway. This tumor‐associated MHC‐II expression phenomenon has been observed in various tumors, including melanoma, breast cancer, colorectal cancer, ovarian cancer, prostate cancer, classical Hodgkin lymphoma, glioma, and non‐small cell lung cancer [36]. The level of MHC‐II expression has been significantly correlated with the magnitude of the anti‐tumor immune response and clinical prognosis [37]. According to references, the dysregulation of HDACs is one of the main factors to the reduced expression of MHC I and II antigens. These enzymes remove acetyl groups from histone proteins, resulting in a more condensed chromatin structure, which subsequently inhibits the transcription of MHC I‐ and II‐related genes [38]. LBH589 enhances the transcription of MHC I/II‐related genes through the regulation of HDAC activity, leading to increased histone acetylation levels and a more relaxed chromatin structure. The protein encoded by the *H2‐D1* gene constitutes a component of MHC class I molecules and plays a role in antigen presentation [37, 39]. The results of mRNA sequencing and RT‐PCR both revealed that LBH589 significantly increased the expression of *H2‐D1* gene in 4T1 cells, and the expression of MHC I on the surface of 4T1 cells increased from 0.11% ± 0.05% to 3.71% ± 0.39% (72 h) (Figures 3D and 5F). In addition, LBH589 significantly increased the expressions of *H2‐Aa*, *H2‐Eb1*, and *CD74* related to MHC II in 4T1. The expression of MHC II on the surface of 4T1 cells increased from 0.25% ± 0.02% to 12.59% ± 1.15% (72 h) (Figures 3D and 5F). The proteins encoded by *H2‐Aa* and *H2‐Eb1* genes, as MHC II molecules, play a role upstream or internally in antigen processing and the presentation of exogenous peptide antigens by MHC II, and have a positive regulatory effect on T cell differentiation. *CD74* facilitates the presentation of exogenous antigenic peptides by MHC II molecules, thereby contributing to the initiation of specific humoral immune responses [40]. LBH589 upregulates the expression levels of *H2‐Aa, H2‐Eb1, and CD74* genes, thereby promoting the presentation of pathogen‐derived antigens by MHC II molecules to CD4^+^ T cells and facilitating the activation of immune responses. In summary, LBH589 significantly promoted the expression of MHC I/II‐related genes in 4T1 cells, enhanced the expression of MHC I/II molecules on the surface of 4T1 cells, and thereby improved cellular immunity. The results of BMDCs co‐culture showed that 4T1 cells treated with LBH589 had a stronger ability to promote the maturation of BMDCs, which was conducive to presenting antigen‐specific signals to T cells (Figure 5D,E).

The results of animal experiments confirmed that LBH589 not only induces apoptosis of 4T1 cells at the cellular level, but also enhances anti‐tumor immune responses in vivo, thereby significantly inhibiting tumor growth. Immune activation assays further demonstrated that LBH589 treatment significantly activated anti‐tumor immunity in vivo. The proportions of CD8^+^ T and CD4^+^ T cells in lymph nodes and spleen were markedly increased, along with a significant rise in the proportion of NK cells in the spleen (*p* < 0.05, Figure 7). Notably, although the CD4^+^ T cells rate increased, this subset includes immunosuppressive cells, such as Treg and Th17 cells. However, based on the previously mentioned mechanism by which MHC II primarily activates CD4^+^ T cells with anti‐tumor activity, and supported by the observed inhibition of tumor growth and activation of immune responses in vivo, it is hypothesized that the increased CD4^+^ T cells are mainly anti‐tumor CD4^+^ T cell subtypes. Of course, further investigation is still needed to determine the specific subtypes of the increased CD4^+^ T cell population.

The serum levels of inflammatory cytokines demonstrated that the levels of MCP‐1, IL‐1β, IL‐17, and IL‐10 were significantly reduced, whereas the level of IFN‐γ was markedly elevated (*p* < 0.05, Figure 8). These changes in inflammatory cytokines are mainly attributed to the immunostimulatory effects of LBH589, which promotes the secretion of anti‐tumor cytokines while suppressing the production of cytokines that promote tumor progression. IFN‐γ is a tumor‐suppressive cytokine produced by anti‐cancer activity immune cells such as Th1 cells, CD8^+^ T cells, and NK cells. The observed increase in IFN‐γ levels aligns with the significant increase in CD8^+^ T cells and NK cells in the lymph nodes and spleen of treated mice. IL‐10 is predominantly secreted by immunosuppressive cells, such as Treg cells, and functions as an immunosuppressive cytokine. Therefore, the decrease of IL‐10 levels in serum reflects the alleviation of the tumor immunosuppressive microenvironment. MCP‐1 is a key regulatory molecule in the TME participating in the occurrence, progression, and metastasis of tumors through complex mechanisms. It directly promotes the proliferation, migration, and EMT of tumor cells by activating the CCR2 receptor and its downstream PI3K/AKT, MAPK, and other signaling pathways and remodels the immunosuppressive microenvironment by recruiting tumor‐associated macrophages and myeloid‐derived suppressor cells. Furthermore, MCP‐1 can also drive tumor invasion and distant metastasis by inducing angiogenesis, enhancing matrix metalloproteinase activity, and promoting premetastatic niche formation [41, 42, 43]. Thus, decreased MCP‐1 levels may serve as an indicator of alleviated immunosuppression in the tumor microenvironment. Although some studies reported that IL‐1 and IL‐17 may exert inhibitory effects on tumor growth under certain specific conditions, such as acute inflammation and the early phase of immune activation. But in most researches, IL‐1 and IL‐17 promote tumor progression through facilitating the formation of an immunosuppressive tumor microenvironment, enhancing tumor angiogenesis, and increasing the proliferative, migratory, and invasive capacities of tumor cells [44, 45]. Based on the inhibition of tumor growth and the increased infiltration of CD8^+^ T cells and NK cells, the decrease of IL‐1 and IL‐17 levels in serum indirectly suggests an alleviation of the tumor immunosuppressive microenvironment. In addition, HDAC is also a key regulatory factor for the expression of inflammatory genes, and pan‐HDACi is associated with the abnormal expression of various inflammatory factors [46]. The experiments in vitro and in vivo have demonstrated that HDACi can reduce the expression of inflammatory cytokines such as TNF‐α, IL‐1β, and IL‐6 [47]. Inhibition of HDAC6 has also been confirmed to down‐regulate the production of MCP‐1, IL‐6, IL‐1β, and TNF‐α in various mouse disease models [48]. This may also contribute to the reduction in serum levels of MCP‐1 and IL‐1β.

Collectively, all data demonstrate that LBH589 significantly enhances the immunogenicity of 4T1 cells and strengthens anti‐tumor immune responses in vitro and in vivo, effectively remodeling the immunosuppressive tumor microenvironment. Despite the clinical success of immune checkpoint blockade for TNBC, a substantial proportion of patients fail to achieve pathological complete response or exhibit limited responsiveness after multiple lines of therapy. Therefore, strategies that enhance tumor immunogenicity and overcome immune resistance are urgently needed. Our results indicate that LBH589 may play a role as an immunotherapy sensitizer, priming tumors for improved responses to immune‐based treatments. It offers a promising strategy to extend the clinical utility of this established drug as an immunomodulatory agent. Especially, its combination with immune checkpoint inhibitors (e.g., anti‐PD‐1/PD‐L1 therapies) may enhance therapeutic efficacy by simultaneously promoting antigen presentation and alleviating immunosuppression. Future studies should focus on optimizing combination regimens, defining responsive patient populations, and identifying predictive biomarkers to fully realize the potential of LBH589 in cancer immunotherapy.

## 5 Conclusions

Currently, due to the lack of biological specificity, side effects, and inter‐patient variations, the use of HDACi therapy as a standalone therapy remains a challenging goal. This research systematically investigated the mechanism of LBH589 in triple‐negative breast cancer and comprehensively elucidated the distinct therapeutic mechanism of LBH589, which integrates both anti‐tumor activity and immune modulation. On the one hand, LBH589 suppresses tumor cell proliferation and metastasis by down‐regulating *c‐myc* expression, disrupting the EMT process, and inducing apoptosis. On the other hand, it enhances anti‐tumor immunity by up‐regulating genes involved in antigen presentation pathways. These findings not only expand the current understanding of LBH589 mechanisms of action, but also exhibit the potential of LBH589 as an immunomodulatory enhancer, offering a clinically translatable strategy to broaden its therapeutic utility.

## Author Contributions

**Liang Anjing:** methodology, visualization, writing – original draft. **Yuan Rong:** methodology, visualization. **Xiang Su:** methodology. **Cheng Liang:** methodology. **Hou Jue:** methodology. **Chen Zhu:** conceptualization, funding acquisition, writing – review and editing, supervision.

## Funding

This work was supported by the Scientific Research Development Fund of North Sichuan Medical College (CBY23‐ZDA07), the Sichuan Medical and Health Care Promotion Institute Scientific Research Project (KY2024QN0059), the Scientific Research Project of the Sichuan Province Science and Education Promotion Association for the Revitalization (KJXC24‐0307).

## Ethics Statement

Ethical approval was granted by the Ethics Committee of North Sichuan Medical Collage (No. 2024073).

## Conflicts of Interest

The authors declare no conflicts of interest.

## Supporting information


**Figure S1:** The full WB images of Ac‐H3 and Ac‐α‐Tubulin; A: the control group, B: 0.01 μM LBH589, C:0.05 μM LBH589, D:0.10 μM LBH589; *n* = 3.
**Figure S2:** The full WB images of *N‐cadherin, E‐cadherin, Vimentin*; A: the control group, B: LBH589 group; *n* = 3.
**Figure S3:** The full WB images of *c‐myc*; A: the control group, B: LBH589 group; *n* = 3.

## Data Availability

The data that support the findings of this study are available on request from the corresponding author. The data are not publicly available due to privacy or ethical restrictions.
