# Supplementary material for: Dual Role of LBH589 in Triple‐Negative Breast Cancer: Inhibition of Tumor Growth and Enhancement of Antitumor Immunity
Source: Cancer Rep (Hoboken). 2026 May 21;9(5):e70581. doi: 10.1002/cnr2.70581 (PMC13240335; doi:10.1002/cnr2.70581)
Supplement: Supplementary file 1 — Figure S1: The full WB images of Ac‐H3 and Ac‐α‐Tubulin; A: the control group, B: 0.01 μM LBH589, C:0.05 μM LBH589, D:0.10 μM LBH589; n = 3. Figure S2: The full WB images of N‐cadherin, E‐cadherin, Vimentin; A: the control group, B: LBH589 group; n = 3. Figure S3: The full WB images of c‐myc; A: the control group, B: LBH589 group; n = 3. [file CNR2-9-e70581-s001.docx]

**Dual Role of LBH589 in Triple-Negative Breast Cancer: Inhibition of Tumor Growth and Enhancement of Antitumor Immunity**

**Liang Anjing^1,#^, Yuan Rong^1,#^, Xiang Su^1^, Cheng Liang^2^, Hou Jue^1^, Chen Zhu^1,^**^🖂^

^1.^ Institute of Tissue Engineering and Stem Cells, Beijing Anzhen Nanchong Hospital of Captial Medical University & Nanchong Central Hosptial, North Sichuan Medical College, Nanchong 63700, Sichuan, China

^2^ Department of Surgery II, Nanchong Hospital of Traditional Chinese Medicine，Nanchong 63700, Sichuan, China

#: These authors contributed equally to this work as co-first authors.


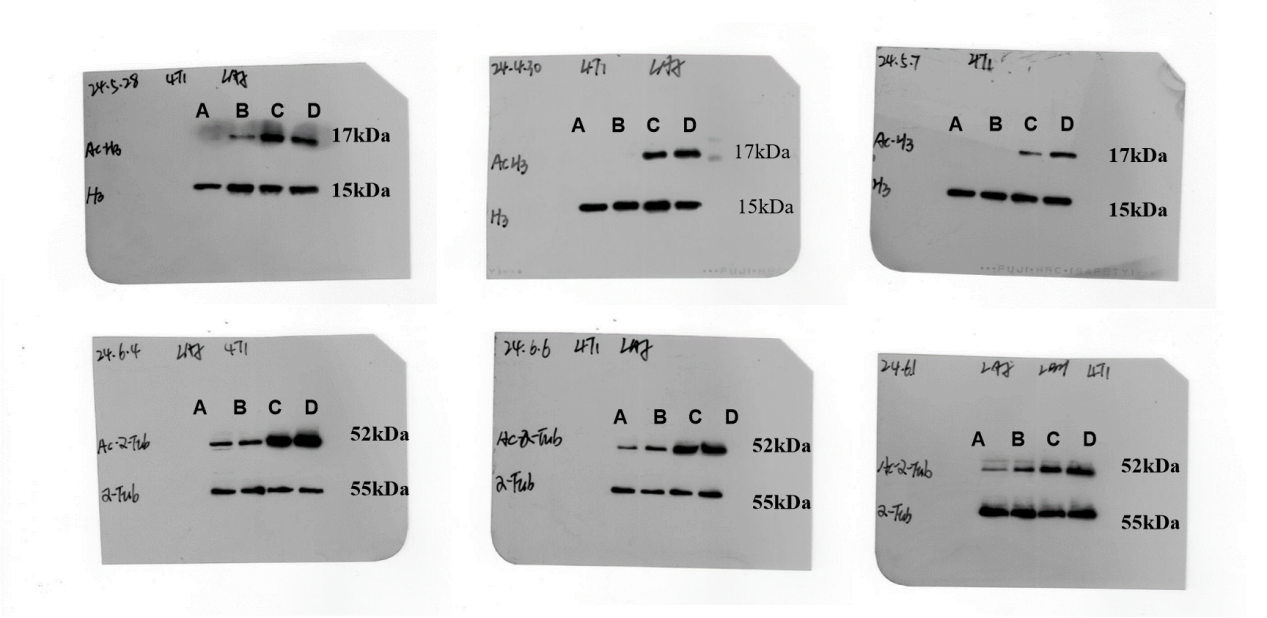


Figure S1.The full WB iamges of Ac-H3 and Ac-α-Tubulin; A: the control group, B: 0.01μM LBH589, C:0.05μM LBH589, D:0.10 μM LBH589; n=3.


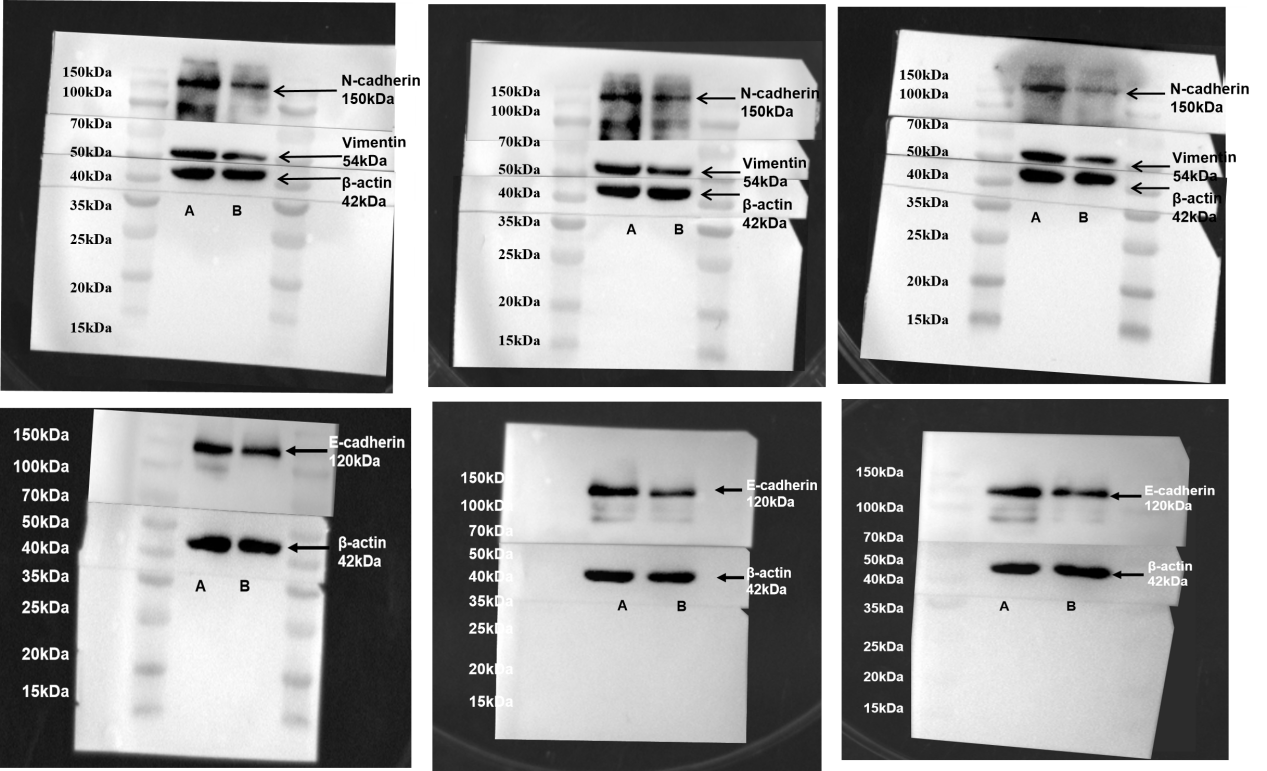


Figure S2.The full WB iamges of *N-cadherin, E-cadherin, Vimentin*; A: the control group, B: LBH589 group; n=3.


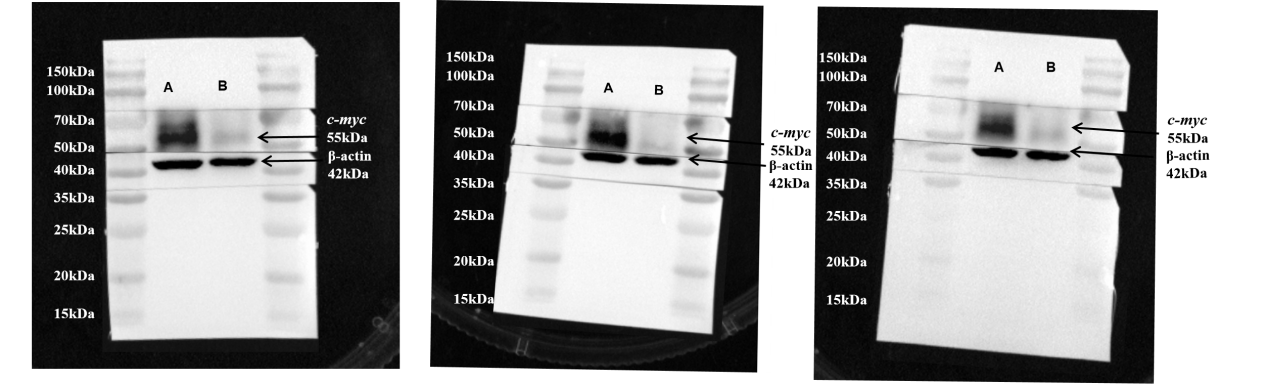


Figure S3.The full WB iamges of *c-myc*; A: the control group, B: LBH589 group; n=3.
